# Supplementary figures and images for: Locations in the Neocortex: A Theory of Sensorimotor Object Recognition Using Cortical Grid Cells
Source: Front Neural Circuits. 2019 Apr 24;13:22. doi: 10.3389/fncir.2019.00022 (PMC6491744; doi:10.3389/fncir.2019.00022)

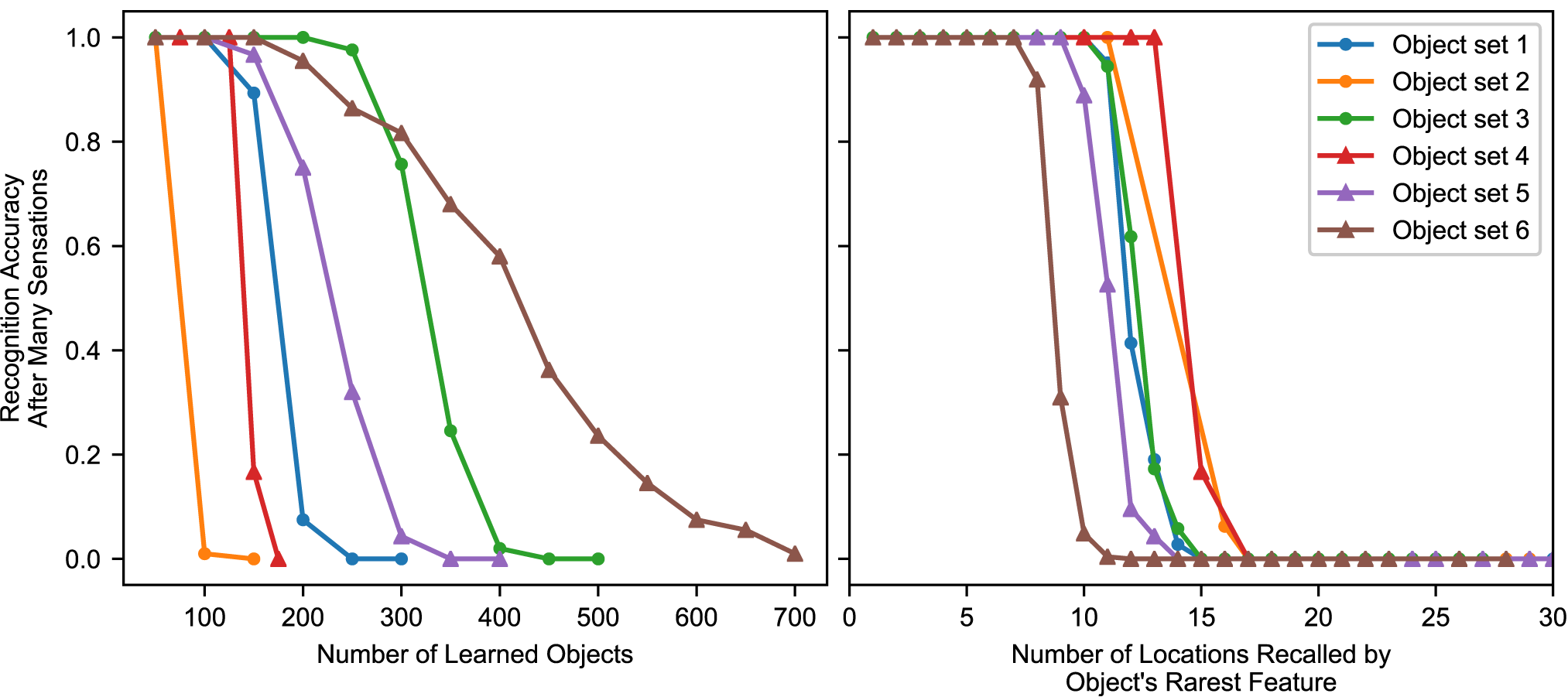

Supplement: FIGURE S1 — Varying the object statistics, the model’s breaking point varies significantly relative to number of learned objects. The breaking point is much more consistent relative to the number of locations recalled by object features. In these charts we use a single model and test on 6 different distributions of objects. The model uses 10 modules with 10 × 10 cells per module. (Left) The network’s capacity depends on the statistics of objects. The network’s performance begins to break down after a certain number of objects, and this breaking point can vary by orders of magnitude with different object distributions. (Right) This breaking point varies significantly less when described in terms of “number of locations recalled by a sensation” rather than “number of objects learned”. Using the same data from the first chart, for each object we measure the total number of occurrences of the object’s rarest feature, and we plot recognition accuracy against this number. With each of these object distributions, the model reaches its breaking point when the number of recalled locations is within a small interval – conservatively, between 7 and 15. There is still some variation due to the statistics of the object’s other features (not just its rarest feature), but the number of occurrences of the rarest feature provides a good first approximation for whether the network will recognize the object. (Object descriptions). Each object set had 100 unique features and 10 features per object, except where otherwise noted. The first three sets generate objects using the same strategy as all the other simulations, varying the parameters. The last three use different strategies. Object Set 1: baseline. Object Set 2: 40 unique features rather than 100. Object Set 3: 5 features per object rather than 10. Object Set 4: Every feature occurs the same number of times, ± 1, rather than each object being randomly selected set of features with replacement. Object Set 5: Bimodal distribution of features, [file Image_1.TIF]
